# Oxidative stress and inflammation in cerebral cavernous malformation disease pathogenesis: Two sides of the same coin

**DOI:** 10.1016/j.biocel.2016.09.011

**Published:** 2016-12

**Authors:** Saverio Francesco Retta, Angela J. Glading

**Affiliations:** a Department of Clinical and Biological Sciences, School of Medicine and Surgery, University of Torino, Regione Gonzole 10, 10043 Orbassano, Torino, Italy; b CCM Italia Research Network^1^; c University of Rochester Medical Center, School of Medicine and Dentistry, 601 Elmwood Ave, 14642 Rochester, NY, USA

**Keywords:** CCM, cerebral cavernous malformation, fCCM, familial form of CCM, sCCM, sporadic form of CCM, NVU, neurovascular unit, ICH, intracerebral hemorrhage, ROS, reactive oxygen species, COX-2, cycloxygenase-2, AJ, adherens junction, EndMT, endothelial-to-mesenchymal transition, TGFβ, transforming growth factor beta, BMP, bone morphogenetic protein, VEGF, vascular endothelial growth factor, KLF, Kruppel-like factor, Cerebrovascular disease, Cerebral cavernous malformation (CCM), CCM1/KRIT1, CCM2, CCM3/PDCD10, Redox signaling, Antioxidant response, Autophagy, Oxidative stress, Inflammation, Angiogenesis, Vascular homeostasis, Blood-brain barrier dysfunction, Vascular permeability

## Abstract

Cerebral Cavernous Malformation (CCM) is a vascular disease of proven genetic origin, which may arise sporadically or is inherited as an autosomal dominant condition with incomplete penetrance and highly variable expressivity. CCM lesions exhibit a range of different phenotypes, including wide inter-individual differences in lesion number, size, and susceptibility to intracerebral hemorrhage (ICH). Lesions may remain asymptomatic or result in pathological conditions of various type and severity at any age, with symptoms ranging from recurrent headaches to severe neurological deficits, seizures, and stroke. To date there are no direct therapeutic approaches for CCM disease besides the surgical removal of accessible lesions. Novel pharmacological strategies are particularly needed to limit disease progression and severity and prevent *de novo* formation of CCM lesions in susceptible individuals.

Useful insights into innovative approaches for CCM disease prevention and treatment are emerging from a growing understanding of the biological functions of the three known CCM proteins, CCM1/KRIT1, CCM2 and CCM3/PDCD10. In particular, accumulating evidence indicates that these proteins play major roles in distinct signaling pathways, including those involved in cellular responses to oxidative stress, inflammation and angiogenesis, pointing to pathophysiological mechanisms whereby the function of CCM proteins may be relevant in preventing vascular dysfunctions triggered by these events. Indeed, emerging findings demonstrate that the pleiotropic roles of CCM proteins reflect their critical capacity to modulate the fine-tuned crosstalk between redox signaling and autophagy that govern cell homeostasis and stress responses, providing a novel mechanistic scenario that reconciles both the multiple signaling pathways linked to CCM proteins and the distinct therapeutic approaches proposed so far. In addition, recent studies in CCM patient cohorts suggest that genetic susceptibility factors related to differences in vascular sensitivity to oxidative stress and inflammation contribute to inter-individual differences in CCM disease susceptibility and severity.

This review discusses recent progress into the understanding of the molecular basis and mechanisms of CCM disease pathogenesis, with specific emphasis on the potential contribution of altered cell responses to oxidative stress and inflammatory events occurring locally in the microvascular environment, and consequent implications for the development of novel, safe, and effective preventive and therapeutic strategies.

## Highlights

• CCM proteins play pleiotropic roles in various redox-sensitive signaling pathways.
• CCM proteins modulate the crosstalk between redox signaling and autophagy that govern cell homeostasis and stress responses.
• Oxidative stress and inflammation are emerging as key focal determinants of CCM lesion formation, progression and severity.
• The pleiotropic functions of CCM proteins may prevent vascular dysfunctions triggered by local oxidative stress and inflammatory events.
• The distinct therapeutic compounds proposed so far for CCM disease share the ability to modulate redox signaling and autophagy.

## Graphical abstract

**Figure:**
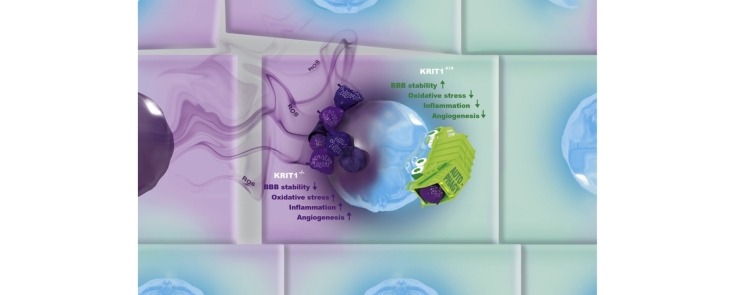

**Towards a unifying mechanism for CCM disease pathogenesis and treatment**.

CCM proteins play pleiotropic roles in distinct redox-sensitive pathways by modulating the fine-tuned crosstalk between redox signaling and autophagy. Effective autophagy removes ROS-generating cellular trash, including damaged mitochondria, to rejuvenate cell environment, thus serving a cytoprotective function for the maintenance of endothelial cell monolayer integrity and functionality and blood–brain barrier (BBB) stability even under adverse stress conditions. Loss-of-function of a CCM protein (e.g., KRIT1) causes defective autophagy and altered redox signaling, affecting BBB stability and sensitizing endothelial cells to local oxidative stress and inflammatory events, which may act as key pathogenic determinants of focal formation and progression of CCM lesions. The common capacity to modulate the interplay between autophagy and redox signaling reconciles the distinct pharmacological approaches proposed so far for CCM disease prevention and treatment.

## 1 Introduction

Cerebral Cavernous Malformations (CCM), also known as cavernous angioma or cavernoma, are vascular malformations consisting of closely clustered, abnormally dilated and leaky capillary channels (caverns) lined by a thin endothelial layer (Batra et al., 2009, Fontanella, 2015, Gault et al., 2004, Rigamonti, 2011). Lesions are devoid of normal vessel structural components, such as pericytes and astrocyte foot processes, but are surrounded by a thick, segmentally layered basal membrane (Clatterbuck et al., 2001). CCM lesions can occur anywhere in the body, but usually produce serious signs and symptoms only when they occur in brain and spinal cord, where they account for 5–15% of all vascular malformations. Retinal, skin, and liver lesions have also been occasionally reported in association with brain lesions. Within the brain, CCM can occur as single or multiple lesions (even hundreds), ranging in size from a few millimeters to a few centimeters. Lesions can remain clinically silent for a lifetime, or unpredictably give rise to various clinical symptoms including headaches, neurological deficits, seizures, stroke, and intracerebral hemorrhage (ICH) (Batra et al., 2009, Fontanella, 2015, Gault et al., 2004, Rigamonti, 2011).

**Fig. 1:**
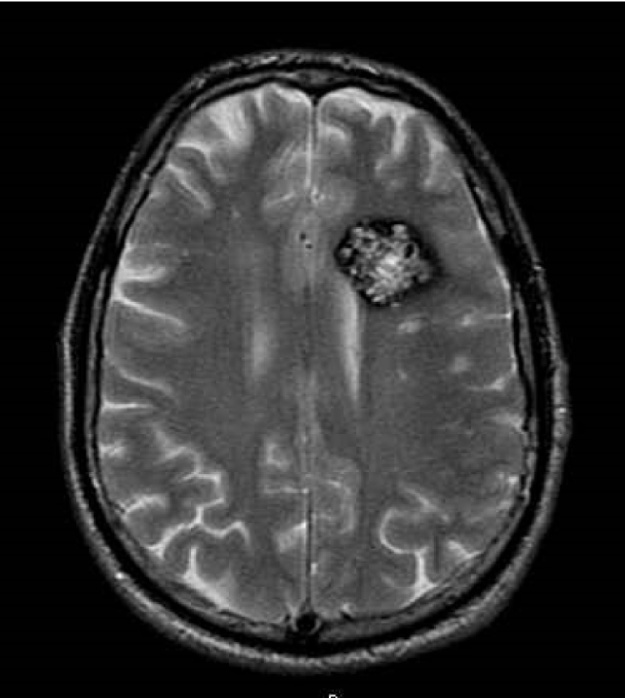
MRI appearance of a Cerebral Cavernous Malformation. Axial T2 FSE MRI of a CCM lesion in an affected patient (*image courtesy of Dr. Maria Consuelo Valentini, “Città della Salute e della Scienza” University Hospital of Torino, Italy*).

Diagnosis is commonly made by magnetic resonance imaging (MRI) (Fig. 1), although detection is far more likely via gradient-echo (GRE) or susceptibility-weighted imaging (SWI), which can unmask small lesions (Campbell et al., 2010, Cooper et al., 2008, de Souza et al., 2008). Because of a large series of MRI and autopsy studies, CCM disease has been recognized as a common clinical entity. Indeed, the prevalence of CCM lesions in the general population has been estimated to be about 0.3%–0.5%, accounting for approximately 24 million people worldwide (Batra et al., 2009, Fontanella, 2015, Rigamonti, 2011). Nevertheless, knowledge and risk awareness of this disease is generally poor. Moreover, diagnosis is mainly possible only when lesions become symptomatic, because the majority of CCM lesions apparently remain clinically and biologically quiescent during most of the host’s lifetime. Indeed, despite the high prevalence of CCM lesions, approximately only 30% of affected people will eventually develop clinical symptoms, which are extremely variable and may have a major impact on the quality of life. The initial presentation of symptoms may occur at any age without sex predominance, although the typical age of onset is between the second and fifth decades of life (Batra et al., 2009, Fontanella, 2015, Rigamonti, 2011).

CCM is a disease of proven genetic origin (OMIM 116860) that may arise sporadically or is inherited as an autosomal dominant condition with incomplete penetrance and variable expressivity (Cavalcanti et al., 2012, Riant et al., 2010). The sporadic form (sCCM) accounts for up to 80% of cases, whereas the familial form (fCCM) accounts for at least 20% of cases. Genetic studies have so far identified three genes whose mutation causes CCM: *KRIT1* (*CCM1*), *MGC4607* (*CCM2*) and *PDCD10* (*CCM3*), which account for about 50%, 20% and 10% of the fCCM cases, respectively. The remaining 20% of cases have been attributed either to other so far undetected genetic alterations in the three known CCM genes or to mutations of a fourth as yet unidentified CCM gene (Choquet et al., 2015, Riant et al., 2010). A genetic founder mutation in *KRIT1* (Q455X, also known as the common Hispanic mutation) is found in descendants of Hispanic-Americans who settled in the southwestern United States and northern Mexico states (Polymeropoulos et al., 1997), and today accounts for the largest population of fCCM worldwide, with thousands of affected patients and varying degrees of clinical severity (Choquet et al., 2014a, Sahoo et al., 1999). Recent studies of this population have facilitated the identification of genetic and environmental risk factors associated with CCM disease progression and severity (Choquet et al., 2014a, Choquet et al., 2014b, Choquet et al., 2016). Like the common Hispanic mutation, most of the hundreds of distinct mutations identified so far in the three known CCM genes are loss-of-function mutations (Choquet et al., 2015, Riant et al., 2010). Notably, while the sporadic form of the illness typically presents as a solitary lesion, the familial form is characterized by the presence of multiple CCM lesions, which are associated with cutaneous and retinal vascular lesions in 9% and 5% of fCCM cases, respectively. Conversely, in contrast to fCCM cases, CCM lesions of sCCM cases are frequently associated with a developmental venous anomaly (DVA), suggesting the possibility of a different developmental mechanism (Meng et al., 2014, Petersen et al., 2010).

Despite the apparent higher disease severity in fCCM cases, up to 70% of mutation carriers remain asymptomatic or minimally symptomatic throughout life. Moreover, a large variability of disease severity is observed even among family members of similar ages carrying the same disease-associated genetic defect, including wide inter-individual differences in lesion number, size and susceptibility to ICH, suggesting that additional factors other than the disease-causing mutation can contribute to CCM disease pathogenesis (Trapani and Retta, 2015). Although significant advances have been made toward understanding the natural history and molecular basis of CCM disease (Batra et al., 2009, Fischer et al., 2013, Fontanella, 2015, Gault et al., 2004, Marchi et al., 2016b, Rigamonti, 2011), the clinical behavior in individual patients, including the development of numerous and large lesions, and the risk of serious complications such as ICH, remains highly unpredictable (Fontanella and Bacigaluppi, 2015). Furthermore, despite the long-held dogma that CCM lesions are congenital and the clear evidence that they may remain clinically and biologically quiescent during the host’s lifetime, there are several instances where their *de novo* formation, increase in size, and recurrent phases of hemorrhage over time has been carefully documented by serial MRI scans (Acciarri et al., 2009, Jung et al., 2003, Yadla et al., 2010). A complete understanding of pathogenic mechanisms and risk factors associated with onset, clinical progression, and severity of CCM disease remains therefore a major clinical and research challenge. This challenge must be met in order to identify new pharmacological therapies and prognostic factors, thus ultimately providing better options for disease treatment and prevention. Indeed, while medications are available to treat some clinical symptoms caused by CCM lesions, including headaches and seizures, to date there are no direct therapeutic approaches for CCM disease, besides the surgical removal of accessible lesions in patients with intractable seizures or recurrent hemorrhage (Fontanella and Bacigaluppi, 2015). In particular, novel pharmacological strategies are specially needed for treating patients with severe symptomatic disease due to inoperable or multiple lesions, as well as for preventing *de novo* formation of CCM lesions and disease progression in susceptible individuals. In addition, the identification of specific risk and susceptibility factors for developing the most severe forms of CCM disease and appropriate biomarkers of disease progression and severity is required to allow accurate risk assessment and more specific diagnosis, thus providing useful insights into predictors of disease outcome, early therapeutic interventions, and surveillance of treatment effects.

In recent years, great progress has been made toward understanding the molecular basis and mechanisms of CCM disease pathogenesis, revealing a stunning complexity (Marchi et al., 2016b). In contrast, little progress has been made in understanding how this complexity could be fully embraced and integrated into a unique model of interconnected mechanisms that may combine to influence the onset, progression, and severity of CCM disease. Indeed, advances from distinct *in vitro* and *in vivo* studies support an important role for either oxidative stress, inflammation or angiogenesis in the pathogenesis of CCM disease (Corr et al., 2012, Goitre et al., 2010, Goitre et al., 2014, Shi et al., 2016, Wustehube et al., 2010, Zhu et al., 2010). Consistently, accumulating evidence demonstrates that loss-of-function of CCM proteins has pleiotropic effects potentially related to the ability of these proteins to regulate multiple molecules and molecular mechanisms involved in angiogenesis, vascular homeostasis, and cell responses to oxidative stress and inflammation, including those that mediate or modulate cell-cell and cell-matrix adhesions, cytoskeleton dynamics, redox signaling, and gene expression (Marchi et al., 2016b). However, despite their well-established interconnected nature, the potential causes and mechanisms of CCM disease are usually considered separately and attributed to specific functions of CCM proteins.

This review intends to bring together current knowledge and ongoing investigations that highlight the pleiotropic effects of CCM proteins in the pathophysiology of CCM disease. This will include their multifaceted roles in adaptive cell responses to oxidative stress, inflammation, and angiogenesis— emphasizing the intricate interplay between these processes. We will also examine to what extent this comprehensive understanding appears promising for the development of novel preventive and therapeutic strategies, and the identification of useful diagnostic and prognostic biomarkers.

## 2 Current knowledge of the molecular basis and mechanisms of CCM disease pathogenesis

Comprehensive analysis of the three known CCM genes in mutation carriers has indicated that CCM protein functions need to be severely impaired for pathogenesis (Riant et al., 2010). Consistently, molecular and immunohistochemical analysis of surgical samples have suggested that CCM lesion genesis requires complete loss-of-function of a CCM gene through a two-hit molecular mechanism (Akers et al., 2009, Gault et al., 2004, McDonald et al., 2011, Pagenstecher et al., 2009). However, the second somatic mutation was detected only in a small percentage of cells (Akers et al., 2009). Moreover, both clinical reports and experiments in animal models have raised the possibility that the ‘second hit’ may not be limited to genetic disruptions but could also take the form of recurrent exposure of the particularly sensitive brain vasculature to local cellular stresses (Jung et al., 2003, Leblanc et al., 2009, Whitehead et al., 2009). Accordingly, it has been reported that CCM3 mouse mutants only develop lesions in conjunction with severe astrocytosis at the lesion site, suggesting that injury of astrocytes may play a role in lesion formation (Louvi et al., 2011), a notion consistent with clinical reports of CCM developing or expanding after physical trauma, injury, or radiation (Cutsforth-Gregory et al., 2015, Louvi et al., 2011). Furthermore and importantly, the development of CCM lesions induced by endothelial-specific conditional knockout of CCM genes in neonatal mice is highly restricted, both spatially and temporally, despite the pan-endothelial deletion of CCM genes (Boulday et al., 2011, Chan et al., 2011, Gibson et al., 2015, Zhou et al., 2016). This clearly demonstrates that the homozygous loss of CCM genes is not fully sufficient to cause CCM lesion formation and disease progression, and suggests the necessary contribution of additional triggers occurring locally, which may include micro-environmental stress factors or brain injuries. Consistently, clinical features of CCM disease, including lesion number and size, rate of recurrent bleeding, age-of-onset, and severity of symptoms, vary greatly even between family members carrying the same causative gene mutation, raising the possibility that the level of risk for CCM disease onset and progression is determined by a combination of multiple factors. These may include disease-associated mutations, distinct microenvironmental stressors, and interindividual variability in stress response (Choquet et al., 2016, Trapani and Retta, 2015).

To date, numerous studies have demonstrated that CCM proteins are implicated in the regulation of major cell structures and signaling mechanisms involved in fundamental physiological functions, as well as in cell responses to various physio-pathological stressors (Marchi et al., 2016b) (Table 1). However, no study has so far undertaken the difficult work of organizing and prioritizing the identified signaling pathways in the context of the natural history of CCM, thus each pathway presently stands as a separate entity, independent of the others. Among others, it has been shown that CCM proteins regulate cadherin-mediated cell-cell junctions (Glading et al., 2007, Glading and Ginsberg, 2010, Lampugnani et al., 2010), integrin-mediated cell-matrix adhesion (Faurobert et al., 2013, Liu et al., 2013, Macek Jilkova et al., 2014), and Rho GTPase-regulated cytoskeleton dynamics (Borikova et al., 2010, Crose et al., 2009, Richardson et al., 2013, Stockton et al., 2010, Whitehead et al., 2009, Zheng et al., 2010).

The initial identification of KRIT1 as a Rap1 GTPase effector that interacts with the endothelial adherens junction (AJ) complex involving VE-cadherin, p120-catenin and β-catenin, and the discovery that loss of this protein promotes the weakening of VE-cadherin-mediated cell-cell adhesion and consequent dysfunction of the endothelial barrier, led to the original hypothesis that loss of blood-brain barrier (BBB) function contributes to the formation of CCM (Glading et al., 2007, Glading and Ginsberg, 2010). Though the interface for this interaction remains unclear, the binding of the small GTPase Rap1 to the FERM domain of KRIT1 is thought to promote the interaction of KRIT1 with the AJ (Glading et al., 2007). Conversely, Liu et al. demonstrated that a mutant KRIT1 that cannot bind Rap1 loses its ability to localize to AJ and remains cytoplasmic (Liu et al., 2011b). Besides Rap1, KRIT1 has other direct binding partners, including CCM2 (Zawistowski et al., 2005) and ICAP1 (Zawistowski et al., 2002, Zhang et al., 2001), and also indirectly binds to CCM3/PDCD10 (Voss et al., 2007). However, while there is evidence that CCM2 is also localized to endothelial cell-cell contacts (Whitehead et al., 2009), this has not been demonstrated for other KRIT1 interacting proteins.

More recently, it has been established that CCM proteins may also regulate integrin-based focal adhesions, which connect endothelial cells to the underlying extracellular matrix, and integrin-mediated signaling (Faurobert et al., 2013, Liu et al., 2013, Macek Jilkova et al., 2014, Renz et al., 2015). Specifically, there is evidence that CCM proteins limit β1 integrin-dependent endothelial cell adhesion, contractility, and fibronectin remodeling by stabilizing ICAP1, an inhibitor of β1 integrin (Faurobert et al., 2013, Liu et al., 2013), and may control endothelial β1 integrin-dependent mechanotransduction in response to shear stress (Macek Jilkova et al., 2014, Renz et al., 2015).

Besides the direct regulation of cadherin- and integrin-mediated cell adhesion, a recurring theme dominating the recent scientific literature is the causal link between loss-of-function of CCM proteins and hyperactivation of the small GTPase RhoA and its effector Rho kinase (ROCK). RhoA activation increases cellular contractility and destabilizes endothelial AJs, thereby reducing endothelial barrier function and increasing vascular permeability (Borikova et al., 2010, Crose et al., 2009, Richardson et al., 2013, Stockton et al., 2010, Whitehead et al., 2009, Zheng et al., 2010). Indeed, the identification of this causal pathogenic mechanism has suggested a potential therapy for CCM disease based on inhibitors of RhoA activation and signaling, including statins and fasudil (Li and Whitehead, 2010, McDonald et al., 2012).

In addition, there is evidence that CCM proteins ensure the quiescence of endothelial cells and inhibit angiogenic responses by either activating the Delta-Notch signaling axis (Schulz et al., 2015, Wustehube et al., 2010, You et al., 2013), inhibiting vascular endothelial growth factor (VEGF) and MAPK/ERK signaling (Cullere et al., 2015, DiStefano et al., 2014, Zhu et al., 2010), or preventing the β1 integrin-dependent activation of a mechanotransduction pathway mediated by the blood flow-sensitive transcription factor Kruppel-like factor 2 (KLF2) (Renz et al., 2015).

Taken together, these results raise the intriguing possibility that CCM proteins orchestrate the signaling crosstalk between integrins and cadherins that coordinately regulates cell–matrix and cell-cell interactions and actin cytoskeleton dynamics involved in the control of vascular morphogenesis and homeostasis, thereby promoting the maintenance of vascular integrity and barrier function. Consistently, the small GTPase Rap1, a major KRIT1 molecular interactor (Glading et al., 2007, Serebriiskii et al., 1997), has been previously reported to play a pivotal role in the signaling network underlying the functional crosstalk between cadherins and integrins (Balzac et al., 2005, Retta et al., 2006).

Loss-of-function of CCM proteins has also been shown to trigger β-catenin and transforming growth factor beta/bone morphogenetic protein (TGFβ/BMP) signaling-driven endothelial-to-mesenchymal transition (EndMT) (Bravi et al., 2016, Bravi et al., 2015, Guan and Couldwell, 2013, Maddaluno et al., 2013), as well as activation of MEKK3 signaling and increased expression of the mechanosensitive transcription factors KLF2 and KLF4 (Cullere et al., 2015, Cuttano et al., 2016, Fisher et al., 2015; Zhou et al., 2015; Zhou et al., 2016). Both of these mechanisms have been suggested to be key determinants of CCM disease pathogenesis (Maddaluno et al., 2013, Bravi et al., 2016, Cuttano et al., 2016, Zhou et al., 2016). Moreover, it has been also suggested that increased Rho activity, as well as proteolytic activity of extracellular matrix metalloprotease ADAMTS, arises secondary to increased MEKK3–KLF2/4 signaling during CCM formation (Zhou et al., 2016). However, while the causal role of these putative downstream effectors remains to be clearly defined (Zhou et al., 2016), there is some inconsistency between recent reports regarding the role of MEKK3 in the regulation of Rho signaling (Fisher et al., 2015, Zhou et al., 2016), as well as the putative relationship between the MEKK3-KLF4 signaling axis and EndMT in disease pathogenesis (Cuttano et al., 2016, Zhou et al., 2016). Furthermore, the functional significance of the contrasting effects of complete (Fisher et al., 2015) and partial (Zhou et al., 2016) endothelial-specific loss of MEKK3 on ICH and brain blood vessels leakage in neonatal mouse models remains ambiguous and open to distinct interpretations (see Section 7).

In this intricate mechanistic scenario, a further level of complexity is added by the discovery that CCM proteins modulate distinct redox-sensitive signaling pathways and mechanisms, including pro-oxidant and antioxidant pathways and autophagy (Gibson et al., 2015, Goitre et al., 2010, Goitre et al., 2014, Guazzi et al., 2012, Marchi et al., 2015), and are implicated in molecular and cellular responses to oxidative stress and inflammatory stimuli (Corr et al., 2012, Goitre et al., 2014). However, besides adding mechanistic complexity, these innovative research findings have also provided a potential integrative explanation for the pleiotropic effects of CCM protein dysfunctions, shedding new light on the mechanisms of CCM pathogenesis and opening new perspectives for disease prevention and treatment (*see Sections below for details*).

In sum, while the overwhelming majority of mechanistic studies have enhanced our understanding of the molecular mechanisms affected by loss-of-function mutations in CCM proteins with consequent implications for the development of distinct potential pharmacological approaches for CCM disease (Table 1), the full clinical impact of this knowledge has so far remained unclear. Indeed, the causal role in CCM pathogenesis and the mechanistic interconnection of the multiple molecules and mechanisms that have been proposed as downstream effectors of CCM proteins remain to be clearly defined. Furthermore, most of these studies have not been able to explain the clear discrepancy between the pan-endothelial loss of CCM proteins and the focal nature of CCM lesions in neonatal mouse models (Boulday et al., 2011, Chan et al., 2011, Gibson et al., 2015, Zhou et al., 2016), leaving open the possibility that additional focal determinants of lesion development are required.

## 3 Redox signaling and oxidative stress: the two emerging faces of CCM protein functions and CCM disease pathogenesis

Structural and functional alteration of brain capillaries, a pathologic hallmark of CCM disease, may reflect both physical and biochemical changes involving all components of the neurovascular unit (NVU) (Girouard et al., 2007, Hawkins and Davis, 2005). This unit consists of cerebral microvascular endothelial cells, astrocytes, pericytes, and extracellular matrix, which constitute the structural basis of the BBB and work together to maintain the integrity of brain capillaries and BBB restrictive features (Clatterbuck et al., 2001). Despite the exact physiological roles of CCM proteins in brain capillaries have not yet been fully defined, several studies in cellular and animal models have demonstrated that endothelial expression of the CCM proteins is crucial for proper angiogenesis and blood vessel structural and functional maintenance, supporting the notion that CCM lesions arise because of primary defects intrinsic to the endothelium (Boulday et al., 2009, He et al., 2010, Liu et al., 2011a, Whitehead et al., 2009, Whitehead et al., 2004, Zhu et al., 2010). However, there is also evidence that either astrocyte or pericyte dysfunctions can contribute to the pathogenesis of CCM disease (Louvi et al., 2011, Schulz et al., 2015, Sweeney et al., 2016), suggesting that CCM lesions may develop as a result of altered structural and functional interactions within the NVU due to the combination of genetic predisposition with a local stressful event.

Among local stress events that might account for a sort of micro-environmental second hit, triggering CCM lesion formation in sensitive NVUs of CCM mutation carriers, there is oxidative stress. This may be caused locally by an imbalance of the physiological equilibrium between the production of reactive oxygen species (ROS), such as superoxide anion (O_2_•^−^), hydrogen peroxide (H_2_O_2_) and organic hydroperoxides, and the ability of cellular antioxidant mechanisms to readily detoxify the reactive intermediates or repair the resulting damage (Goitre et al., 2012, Retta et al., 2012). In particular, the superoxide anion (O_2_•^−^) is the key determinant of the overall effects of ROS, being the precursor of all other major reactive oxygen species found in biological systems, including the powerful oxidants hydroxyl radical (•OH), hydrogen peroxide (H_2_O_2_), and peroxynitrite (OONO^−^) (Goitre et al., 2012, Wolin et al., 2002). Within an intermediate physiological range of concentrations, ROS function as critical signaling molecules implicated in various cell fate decisions and signal transduction pathways, including regulation of cell metabolism, adhesion, migration, proliferation, differentiation and survival (Dodson et al., 2015, Holmstrom and Finkel, 2014). On the other hand, it is well understood that excessively high levels of ROS may cause ‘oxidative stress’ that induces damage to proteins, membrane lipids and nucleic acids, leading eventually to cellular dysfunctions and diseases. Nevertheless, there is increasing evidence that pathological effects can also be evoked by excessively low levels of ROS due to the sustained activation of antioxidant pathways, which may cause a ‘reductive stress' that affects fundamental redox-dependent molecules and molecular mechanisms (Dodson et al., 2015). Thus, the response to ROS displays hormesis, which should be considered when addressing the pathophysiological relevance of ROS in diseases, including vascular diseases, as well as therapeutic approaches with exogenously administered antioxidants or the activation of endogenous antioxidant pathways (Dodson et al., 2015, Johansen et al., 2005).

ROS are generated both constitutively, as common by-products of oxidative metabolism, and in response to the activation of several oxidative enzyme systems. Specifically, the redox complexes I and III of the mitochondrial electron transport chain are the major constitutive source of ROS, converting up to 5% of molecular O_2_ to O_2_•^−^. In addition, ROS are produced by the activity of NADPH oxidase, xanthine oxidase (XO), cytochrome p450 monooxygenase (CYP), uncoupled NO synthase (NOS), myeloperoxidase (MPO), lipoxygenase (LOX), and cyclooxygenase (COX) enzymes, which can be induced by a variety of endogenous and exogenous chemical and physical stimuli (Goitre et al., 2012). Conversely, the burden of ROS production is largely counteracted by a number of constitutive or inducible antioxidant defense systems, whose cytoprotective functions are essential to prevent and counteract oxidative stress, thus maintaining or restoring cellular homeostasis (Espinosa-Diez et al., 2015). In particular, mitochondria, the most powerful source of ROS, are endowed with a heavy presence of non-enzymatic antioxidants and antioxidant enzymes, including the superoxide dismutase 2 (SOD2) enzyme, which plays a major role in antioxidant defenses by catalyzing the dismutation of O_2_•^−^ into H_2_O_2_ and O_2_. In turn, H_2_O_2_ is reduced to H_2_O by the catalase and glutathione peroxidase enzymes (Fukai and Ushio-Fukai, 2011, Goitre et al., 2012). In addition, the recognition of dysfunctional mitochondria and their selective targeting for degradation by a specific mode of autophagy, termed mitophagy, has evolved as a defense mechanism of quality control that avoids the accumulation of damaged, ROS-generating mitochondria, thereby contributing to maintain cellular redox homeostasis (Lee et al., 2012, Scherz-Shouval and Elazar, 2011).

All cellular components of NVUs have been shown to generate ROS. Indeed, it is now well established that physiologic concentrations of ROS are endowed with fundamental signaling properties, which are essential for both normal cellular homeostasis and adaptation to changes of the microenvironmental conditions within the NVU. This signaling capacity is mainly due to the reversible oxidation of specific redox-sensitive molecular targets, which act as components of redox-sensitive signal transduction pathways to fulfill a wide range of essential biological functions (Goitre et al., 2012). In contrast, abnormal levels of ROS may exert very damaging effects through oxidative stress, which can result in pathological oxidative modifications affecting cellular components of the NVU and the BBB (Chrissobolis et al., 2011). Pro-oxidant factors and oxidative stress may be produced at NVUs following local metabolic and biochemical changes, inflammatory responses, impaired neurovascular coupling, and ischemia/hypoxia events, as well as by exogenous oxidative insults, including cell exposure to xenobiotics or ionizing radiation, resulting in BBB dysfunction (Chrissobolis et al., 2011, Freeman and Keller, 2012, Goitre et al., 2012). There is now a wealth of evidence indicating that oxidative stress is indeed a major cause of vascular remodeling and NVU dysfunction associated with cerebrovascular diseases (Chrissobolis et al., 2011, Faraci, 2011, Fraser, 2011). In particular, oxidative stress has been clearly implicated in all the major molecular and cellular alterations related to CCM diseases, including destabilization of endothelial cell-cell junctions, increased β1 integrin activation, reduced cellular ability to maintain a quiescent state, increased vascular permeability, and angiogenic activity (Fraser, 2011, Fukai and Ushio-Fukai, 2011, Goitre et al., 2012, Ushio-Fukai, 2009), suggesting that it may represent a significant triggering factor involved in the initiation and progression of CCM disease. Consistently, CCM proteins have been involved in protecting cells against oxidative stress (Fidalgo et al., 2012, Goitre et al., 2010, Goitre et al., 2014), raising the possibility that CCM lesions may result from an impaired oxidative stress defense in microvascular districts of genetically predisposed subjects, and opening new therapeutic perspectives (Gibson et al., 2015, Goitre et al., 2010, Goitre et al., 2014, Moglia et al., 2015, Moglianetti et al., 2016).

Specifically, it was originally demonstrated that KRIT1 plays an important role in maintaining intracellular ROS homeostasis, thereby limiting molecular and cellular oxidative dysfunctions and preserving cellular resistance to oxidative stress (Goitre et al., 2010). This antioxidant and cytoprotective role was associated with the KRIT1-dependent maintenance of mitochondrial homeostasis and modulation of master regulators of cell responses to oxidative stress, including the transcriptional factor FoxO1, its downstream target and major cellular antioxidant enzyme SOD2, and Sirt1 (Goitre et al., 2010). These original findings have been then substantiated by the identification of a novel molecular interactor of KRIT1, the Kelch family protein Nd1-L, which was previously implicated in the regulation of both Rho GTPases and cellular responses to oxidative stress (Guazzi et al., 2012), as well as by the discovery that also CCM3/PDCD10 plays a role in protecting cells from oxidative stress (Fidalgo et al., 2012). In particular, PDCD10 is required for the activation of the GCKIII kinase Mst4 and its relocation to the cell periphery after oxidative stress, where it phosphorylates and activates ezrin/radixin/moesin (ERM) proteins, thereby promoting cell survival (Fidalgo et al., 2012). Furthermore, these findings have been subsequently consolidated and extended by novel findings showing that KRIT1 may exert a protective role against oxidative stress by limiting pro-oxidant and pro-inflammatory pathways, including JNK/c-Jun-dependent redox signaling, and consequent molecular and cellular dysfunctions (Goitre et al., 2014). Indeed, it has been demonstrated that KRIT1 loss-of-function causes the up-regulation of c-Jun, a basic component of the dimeric redox-sensitive transcription factor AP-1, both in cellular models and human CCM tissue samples; conversely, this up-regulation can be reversed by either KRIT1 re-expression or ROS scavenging with antioxidant compounds (Goitre et al., 2014). Notably, overexpression of KRIT1 prevented forced up-regulation of c-Jun induced by oxidative stimuli, suggesting an essential, dose-dependent role for KRIT1 in protecting cells against exogenous oxidative insults by limiting c-Jun-dependent redox pathways (Goitre et al., 2014). In accord with these findings, there is clear evidence that the redox-sensitive transcription factor c-Jun is up-regulated in response to either oxidants or oxidative stress (Kunsch and Medford, 1999, Lee et al., 1996). c-Jun is also associated with vascular dysfunction, including enhanced vascular remodeling and permeability, and inflammatory responses (Lum and Roebuck, 2001, Rojas et al., 2006), as well as with pathological angiogenesis and microvascular diseases in humans (Fahmy et al., 2006, Folkman, 2004, Zhang et al., 2004, Zhang et al., 2006). Interestingly, the up-regulation of c-Jun consequent to KRIT1 loss-of-function is accompanied by the induction of cycloxygenase 2 (COX-2), a major oxidative stress biomarker and inflammatory mediator involved in vascular dysfunctions (Hsieh et al., 2012, Ushio-Fukai and Nakamura, 2008), raising the possibility that KRIT1 loss-of-function might be implicated in synergistic oxidative stress and inflammatory responses (Goitre et al., 2014). Indeed, consistent with a potential role for inflammatory processes in CCM disease pathogenesis, mice heterozygous for the deletion of the KRIT1 gene (*KRIT1*^+/−^) exhibit an enhanced sensitivity to inflammatory stimuli (Corr et al., 2012), and there is evidence that inflammatory response occurs in CCM lesions (Shenkar et al., 2007, Shi et al., 2009) (see Sections 4, 5 and 7).

Both the original findings linking KRIT1 to the control of redox-sensitive signaling pathways, and the original hypothesis that loss-of-function mutations of CCM genes may predispose endothelial cells to oxidative stress-mediated dysfunctions (Goitre et al., 2010, Goitre et al., 2014) have gained strong support from a recent integrated and cooperative research approach based on cellular and animal models of loss-of-function of CCM2 (Gibson et al., 2015). In particular, this study demonstrated that CCM2 loss-of-function in endothelial cells induces increased ROS and decreased FoxO1 expression (Gibson et al., 2015), reflecting physiopathologic mechanisms common to KRIT1 (Goitre et al., 2010), and provided evidence that oxidative stress may be a driving force in CCM at the level of individual cells (Gibson et al., 2015). Importantly, this study suggested also new promising preventive and therapeutic options based on two available repurposed drugs: Tempol, a SOD-mimetic ROS scavenger (Pires et al., 2010), and cholecalciferol (vitamin D3), a physiological compound endowed with autophagy-inducing and antioxidant properties that has been shown to exert protective effects against oxidative injury in various cell types, including vascular endothelial cells, neurons and glial cells (Garcion et al., 2002, Hoyer-Hansen et al., 2010, Ting and Lee, 2013, Wiseman, 1993). In particular, cell treatment with these compounds was effective in reversing the hyperactivation of RhoA and consequent biological outcomes induced by CCM2 knockdown, including actin stress fiber formation, adherens junction weakening, and endothelial barrier dysfunction (Gibson et al., 2015). Moreover, both these compounds showed an enhanced effectiveness in decreasing lesion burden in a mouse model of CCM disease (Gibson et al., 2015), as compared to the previously reported putative therapeutic benefits of statins (Li and Whitehead, 2010, Whitehead et al., 2009).

In this light, the reported role of the TGF-β pathway in CCM disease pathogenesis (Maddaluno et al., 2013) might be connected to, and downstream of deregulation of oxidative stress and c-Jun activity. Indeed, there is clear evidence that ROS can stimulate the activation of the TGF-β pathway with important consequences on cellular functions (Fukawa et al., 2012, Gonzalez-Ramos et al., 2012), and it has also been demonstrated that this ROS-mediated regulation is dependent on c-Jun transcriptional activity (Gonzalez-Ramos et al., 2012). Furthermore, the established functions of CCM proteins in the regulation of both cadherin- and integrin-mediated cell adhesion and RhoA-dependent actin cytoskeleton dynamics might be directly related to the emerging important role of ROS and redox signaling in the modulation of the functional crosstalk between cadherins, integrins and small GTPases (Ferro et al., 2012, Goitre et al., 2012). In particular, it is intriguing that the activation of redox signaling complexes at integrin-mediated cell-matrix adhesion sites and cadherin-mediated cell-cell junctions induces opposite effects, leading to assembly of integrin-mediated focal adhesions and disassembly of cadherin-mediated AJs, respectively, followed by morphological transition toward a mesenchymal phenotype (Goitre et al., 2012), which recapitulate almost all the major molecular mechanisms of CCM pathogenesis. Finally, given the well-established role of MEKK3 and KLFs as both targets and regulators of redox signaling and cellular responses to oxidative stress (Chen et al., 2015, Hamik and Jain, 2012, Son et al., 2011), also the recently reported involvement of the MEKK3-KLF4 signaling axis in CCM pathogenesis (Cuttano et al., 2016, Zhou et al., 2016) might imply dysregulation of redox signaling and oxidative stress (see Sections 5 and 7).

Notably, the findings described above provide also an alternative explanation to the previously suggested effectiveness of statins (e.g., simvastatin) (Li and Whitehead, 2010), fasudil (McDonald et al., 2012), and sulindac derivatives (Bravi et al., 2015) as potential therapy for CCM disease. Indeed, both statins and fasudil have been shown to reduce the expression and transcriptional activity of c-Jun (Dichtl et al., 2003, Wang et al., 2011a, Wang et al., 2011b), and there is evidence that the Rho GTPase pathway can be directly activated by ROS (Aghajanian et al., 2009, Ferro et al., 2012). Moreover, both statins, fasudil and sulindac derivatives are known to exert powerful antioxidant activities in endothelial cells, including inhibition of superoxide production and improvement of ROS scavenging, which in some cases was even more effective than endogenous antioxidants such as GSH (Adam and Laufs, 2008, Costa et al., 2005, Kuhlmann et al., 2008, Ma et al., 2011).

Recent findings have also linked defective autophagy to enhanced endothelial cell sensitivity to oxidative stress, which suggests a pathogenetic mechanism that reconciles both the pleiotropic functions of CCM proteins and the distinct therapeutic approaches proposed so far (Marchi et al., 2015, Marchi et al., 2016a, Marchi et al., 2016b). Indeed, it is well established that autophagy inducers limit ROS accumulation and oxidative stress by stimulating the autophagic degradation of ROS-generating mitochondria (Lee et al., 2012, Scherz-Shouval and Elazar, 2011). On the other hand, there is compelling evidence that antioxidant compounds, such as those indicated above, can induce autophagy, including statins (Andres et al., 2014, Wei et al., 2013, Zhang et al., 2013a), fasudil (Iorio et al., 2010), sulindac derivatives (Chiou et al., 2011, Gurpinar et al., 2013), and vitamin D3 (Hoyer-Hansen et al., 2010, Kim et al., 2012, Lisse and Hewison, 2011, Wu and Sun, 2011). Together with recent findings suggesting that the interplay between defective autophagy and redox imbalance may be integral to the development and progression of CCM lesions by sensitizing endothelial cells to local oxidative stress events (Marchi et al., 2015), these observations point to autophagy as a major redox-sensitive mechanism that justifies the reported effectiveness of the different potential therapeutic compounds described so far (Marchi et al., 2016a, Marchi et al., 2016b).

Taken together, these considerations indicate that CCM proteins are new players in redox biology, and suggest that oxidative stress may contribute significantly to the development and progression of CCM disease by influencing molecular and cellular mechanisms underlying its major phenotypic hallmarks, including destabilization of endothelial cell-cell junctions, increased β1 integrin activation, increased vascular permeability, and enhanced angiogenic and inflammatory responses. In addition, oxidative stress is also a likely candidate to drive progression and severity of CCM disease. Indeed, several genes encoding proteins involved in ROS metabolism and vascular responses to oxidative stress are characterized by single nucleotide polymorphisms (SNPs) that confer substantial inter-individual variability in susceptibility to various oxidative stress-related pathologies, including vascular diseases (Trapani and Retta, 2015). Consistently, a recent study by Choquet et al. identified oxidative stress-related genetic modifiers, including genetic variants in cytochrome P450 mono-oxygenase (CYP) and extracellular matrix metalloproteinase (MMP) genes, that influence the severity of fCCM disease as manifested by ICH and greater total or large lesion counts (Choquet et al., 2016), suggesting that inter-individual variability in susceptibility to oxidative stress may contribute to CCM disease pathogenesis and severity.

Overall, the evidence accumulated so far weighs heavily in favor of a unifying pathogenic mechanism whereby the loss-of-function of CCM proteins sensitize vascular cells to local oxidative stress events, and raises the possibility that inter-individual variability in susceptibility to oxidative stress may contribute to CCM disease pathogenesis.

## 4 CCM proteins regulate the inflammatory response

The idea that susceptibility to microenvironmental elements and oxidative stress can be conferred to cells by loss-of-function mutations in CCM proteins suggests that these proteins may also play a role in limiting the endothelial response to inflammation. Indeed, as stabilizers of endothelial AJs, CCM proteins are optimally placed to regulate the vascular response to inflammation, as the stability of endothelial cell-cell contacts is a key component of vasogenic edema and leukocyte extravasation. Specifically, destabilization or increased turnover of endothelial cell-cell contacts increases the net flux of fluid across the endothelial barrier. This, in turn, carries with it small molecules and proteins that disrupt the osmotic balance between blood and tissue, causing further fluid flux across the vessel wall. At the tissue level, once the flux of fluid into the tissue exceeds the ability of the lymphatic vessels to compensate, fluid accumulates in the tissue, causing edema (Lee and Liles, 2011). KRIT1 heterozygous animals, which appear phenotypically normal, in fact demonstrate increased flux of fluid/protein out of their vessels at a rate more than double of their wildtype littermates. However, these mice do not appear edematous, presumably due to the compensation in fluid management by the lymphatic vessels (Corr et al., 2012). These animals also develop more severe reactions to inflammatory stimuli, and in fact exhibit increased sensitivity to auto-antigens and foreign polysaccharides, demonstrating clearly that loss of KRIT1 decreases the threshold at which an inflammatory response will occur (Corr et al., 2012). The increase in baseline fluid flux has also been observed in fCCM patients, which exhibit increased vascular permeability throughout their brain tissue, not only in areas containing CCM lesions (Girard et al., 2016, Hart et al., 2013). Mechanistically, the underpinnings for an enhanced inflammatory response are also present in human patients; therefore it is highly likely that increased sensitivity to inflammatory stimuli also occurs in human patients.

The role of CCM proteins in the vascular response to inflammation has only recently been investigated, and is one of the only functions for CCM proteins described under non-pathological conditions. Existing data points to an anti-inflammatory role for CCM proteins, in which they function to suppress the activation of, or response to, pro-inflammatory signals. As an effector of Rap1, KRIT1 mediates the ability of this GTPase to stabilize endothelial cell-cell contacts, as has been shown in response to thrombin (Glading et al., 2007) and acute lung injury (Meliton et al., 2015), thus limiting the inflammatory response. Less direct evidence is available regarding the ability of CCM2 and PDCD10 to regulate the endothelial inflammatory response. However, PDCD10 has an emerging role in the hematopoietic system, where it has been shown to play a role in neutrophil degranulation and malignant T-cell survival (Lauenborg et al., 2010, Zhang et al., 2013b). Indeed, KRIT1, CCM2, and PDCD10 are all widely expressed, including in multiple hematopoietic lineages, pericytes and smooth muscle cells, and epithelial cells (Glading and Ginsberg, 2010). Thus the role for these proteins and their requisite complex in inflammation is likely to expand as more studies are performed.

## 5 Role of inflammation in CCM pathogenesis

The pathogenesis of CCM is thought to require genetic loss of both alleles of a single CCM protein, which leads to abnormal endothelial responses, including changes in cell adhesion, cytoskeletal rearrangement, transcriptional activity, and oxidative stress. This is supported by genetic animal models, in which loss of both alleles of KRIT1, CCM2, or CCM3 is required for the formation of CCM-like vascular lesions (Boulday et al., 2009, Chan et al., 2011). However, the large variation in disease severity among family members carrying the same disease-associated genetic defect suggests that additional factors other than the disease-causing mutation can contribute to CCM disease pathogenesis. Several lines of evidence suggest that inflammation may play one or more roles in the etiology of cerebral cavernous malformations and provide a site-specific, local stimulus for lesion formation or progression.

First, partial loss of CCM proteins increases intracellular ROS levels and oxidative stress (Goitre et al., 2010), which contribute to the up-regulation of inflammatory transcription factors including NF-κB, AP-1, and PPAR-γ, leading to the production and release of cytokines and chemokines (Corr et al., 2012). Second, loss of endothelial cell-cell contact, even in heterozygous patients and animals, would likely predispose the vasculature to chronic, low-grade inflammation due to intermittent exposure of the vascular basement membrane, a potent inflammatory stimulus (Mackel et al., 1982). A related example of such a mechanism is observed in radiation-induced cavernous malformation (RICM) in which radiation-induced endothelial damage leads to the stimulation of pro-inflammatory signaling, the development of large, often multiple, vascular malformations that closely resemble CCMs, and hemorrhage (Cutsforth-Gregory et al., 2015, Nimjee et al., 2006, Park et al., 2011). Third, recent evidence suggests that innate immune cells contribute to the vascular permeability phenotype of KRIT1 deficient mice. Transplantation of wildtype bone marrow into endothelial KRIT1 knockout animals restored vascular permeability in these animals to wildtype levels. However, the converse transfer of knockout bone marrow to wildtype animals did not decrease barrier function, suggesting that loss of KRIT1 expression in both the endothelium and the hematopoietic cell compartment is required for the phenotypic effects of KRIT1 depletion (Corr et al., 2012). As the interaction between circulating immune cells and the endothelium in KRIT1 deficient mice appear normal, this suggests that loss of KRIT1 may alter the signaling capacity of hematopoietic cells in some other way, such as through increasing the frequency of the oxidative burst. Interestingly, CCM formation is associated with the increased expression of specific human leukocyte antigen types (Sanus et al., 2007), suggesting that there may also be a genetic contribution from the immune system that predisposes to CCM formation. Finally, while the primacy of inflammatory signaling to CCM formation has yet to be formally established, it is important to note that the wide variety of molecular mechanisms attributed to CCM formation, including down-regulation of cell-cell contacts, altered cell-matrix adhesion, activation of RhoA signaling and cytoskeleton contractility, endothelial to mesenchymal transition, altered expression levels and activity of various transcription factors, increased oxidative stress, and angiogenesis, all fit comfortably under the umbrella of known inflammatory signaling mechanisms. For these reasons, inflammation is a prime candidate for the putative “microenvironmental hit” required for local CCM lesion formation, and in fact, further examination of the role of inflammation in CCM may finally lead to a cohesive mechanism of lesion development.

In recent years, several downstream pathways have been identified which are up-regulated in fCCM, or following loss of one or more of the CCM proteins in mice, as we briefly summarized in Section 2 and Table 1. Conspicuously, of those pathways that have been pharmacologically targeted *in vivo* for their effects on lesion development, none have been able to provide more than 50% relief of lesion burden, suggesting that each pathway is only partially responsible for the CCM phenotype. Notably, significant crosstalk between the inflammatory response and many of these pathways is already suggested by findings in other fields. Down-regulation of cell-cell contacts and activation of RhoA signaling and cytoskeleton contractility (Glading et al., 2007, Stockton et al., 2010) occurs in the absence of CCM proteins, but is also considered a common downstream consequence of pro-inflammatory cytokine signaling and contributes the most strongly to increases in vascular permeability. Increased permeability can contribute to the activation of junctionally-regulated transcriptional factors, such as β-catenin (Glading and Ginsberg, 2010) and Kaiso (unpublished data), both seen in the absence of CCM proteins. These can then upregulate both pro-inflammatory and pro-angiogenic signals, such as VEGF expression (DiStefano et al., 2014), which regulate cellular levels of oxidative stress (Kim and Byzova, 2014). Altered cell-cell and cell-matrix adhesion are hallmarks of EndMT, observed in both CCM protein-deficient cell culture models (Maddaluno et al., 2013) and human CCM lesions (Bravi et al., 2016), which also leads to the upregulation of a host of transcription factors, including KLFs that contribute to the mesenchymal phenotype, but also regulate -and are regulated by- oxidative stress (Chen et al., 2015, Zucker et al., 2014). Recent attention has focused on the activation of MAP kinase signaling, particularly downstream of the scaffold MEKK3, which is activated in mouse models of CCM, and which binds to CCM2 (Fisher et al., 2015, Uhlik et al., 2003). Deletion of mouse MEKK3 leads to loss of neurovascular integrity in neonates (Fisher et al., 2015), but conditional deletion of MEKK3 in CCM2 knockout animals prevents CCM-like lesion formation by blocking activation of KLF4 (Zhou et al., 2016). Notably, MEKK3 is regulated by ROS (Son et al., 2011), and is part of the NF-κB regulatory machinery downstream of pro-inflammatory Toll-like receptors (Qin et al., 2006) and TNF receptors (Blonska et al., 2005), suggesting that activation of MEKK3 signaling could be part of the inflammatory response. KLF2 and KLF4 are also tightly associated with endothelial redox and inflammatory balance. Indeed, both KLFs confer vascular protection via regulation of gene programs that result in an anti-inflammatory, anti-coagulant, anti-adhesive, anti-oxidant state of the endothelium, thus serving as guardians of endothelial health against various stress conditions, including oxidative stress and inflammation (Hamik and Jain, 2012). More specifically, these cytoprotective transcription factors are involved in a complex network of redox-sensitive mechanotransduction signaling that inhibits oxidative stress and inflammation while promoting vascular homeostasis (Chen et al., 2015, Hamik and Jain, 2012), suggesting that these mechanisms could play a role in CCM disease pathogenesis (see Section 7). Clearly, there are many tantalizing hints as to how these pathways work together to promote the development and progression of CCM, yet there is a large amount of work to be done to delineate those connections.

The idea that inflammation could significantly contribute to the formation of *de novo* lesions is attractive for several reasons. Chronic inflammation is known to stimulate a proliferative and angiogenic phenotype, such as that observed in the pannus of rheumatoid arthritis (Szekanecz et al., 2005). Consistently, heterozygous KRIT1 mouse embryos and human CCM lesions exhibit increased endothelial proliferation (Shenkar et al., 2005, Whitehead et al., 2004). Consequently, enhanced proliferation could promote the further loss of CCM protein expression due to amplification of the innate error rate- the putative 2nd hit. Inflammatory signaling also causes changes in gene expression that stimulate fibrosis, both at the level of endothelial extracellular matrix (ECM) secretion and downstream of EndMT (Moonen et al., 2015). Fibrotic changes, which are commonly observed in CCM lesions of mice and humans (Bicer et al., 2010, Clatterbuck et al., 2001, Faurobert et al., 2013), promote EndMT and endothelial proliferation and could also provide a secondary signal driving down-regulation of cell-cell contacts, increased proliferation, and migration due to altered signaling from the ECM. Lastly, the focused nature of inflammation, which is typically confined to a local area, could provide an explanation for the focal nature of CCM. As described above, in genetic animal models in which loss of CCM proteins occur in over 80% of endothelial cells as measured by Cre-mediated recombination, lesions comprise <5% of the total vascular bed (Chan et al., 2011). Furthermore, human CCM tissues only contain a second somatic mutation in 10–15% of cells (Akers et al., 2009), suggesting that lesion development may involve non-cell autonomous epigenetic signaling events. Thus inflammation/inflammatory signaling could help drive lesion formation by promoting a cellular context in which genetic events could drive changes in the local neurovascular environment that lead to endothelial proliferation, reduced vessel integrity, and ultimately distorted vascular morphology.

Inflammation is also a likely candidate to drive progression of CCM lesions. Overwhelming evidence suggests that CCM lesions are highly dynamic, and can grow and shrink in size as well as vary in hemorrhage rate and severity (Acciarri et al., 2009, Jung et al., 2003, Yadla et al., 2010). A recent study by Choquet et al. identified common genetic variations in inflammatory and immune response pathways in patients with fCCM that correlated with hemorrhage, lesion size and lesion number, strongly suggesting that genetic variation in the inflammatory response can influence disease severity and variability (Choquet et al., 2014b). Indeed, the connection between inflammation and hemorrhage is well established in multiple cerebrovascular disorders, including cerebral ischemia (Ahmad et al., 2014), congenital and trauma-induced aneurysm (Kataoka, 2015), cerebral arteriovenous malformation (AVM) (Mouchtouris et al., 2015), and cerebral amyloid angiopathy (Castro Caldas et al., 2015). Anti-inflammatory treatment of these conditions reduces bleed rate and severity. However it is difficult, even in these cases, to distinguish between inflammation preceding the hemorrhagic event, and the inflammatory response generated by the hemorrhage. Thus in CCM lesions, where there is direct evidence of the presence of an innate inflammatory response within large, mature lesions, it remains unclear when the inflammatory response occurred during the natural history of the lesion. Nevertheless, it is clear that inflammatory cells accumulate within CCM lesions and therefore may significantly contribute to disease phenotype (Shi et al., 2009). Curiously, CCM lesions accumulate mast cells, T- and B-cells, in addition to the neutrophils and macrophages that are more typical of the acute inflammatory response (Hagiwara et al., 1999, Shi et al., 2009). Indeed, infiltration of neutrophils and macrophages are common to many cerebrovascular disorders, such as AVM (Chen et al., 2008); however, the accumulation of T-cells- and particularly B-cells- is relatively uncommon, and is more often seen in chronic inflammatory conditions involving the auto-immune response, such as rheumatoid arthritis and multiple sclerosis (Alunno et al., 2015, Ireland et al., 2015). Multiple studies by Shi et al. have identified the presence of oligoclonal B- and plasma cell populations within some CCM lesions (Shi et al., 2007). More recently, they have shown that B-cell depletion can reduce the progression of CCM-like lesions *in vivo* (Shi et al., 2016). While the antigen or antigens recognized by these cellular infiltrates remains unknown, their presence presents a striking parallel to the ectopic B-cell clonal expansion seen in some forms of multiple sclerosis (Owens et al., 2003), raising the possibility that chronic, auto-immune mediated inflammation contributes to the formation or progression of CCM, and that mitigating this destructive influence could be an effective form of treatment for this disease.

Clearly, the hypothesis that inflammation contributes to the development of CCM is worthy of careful consideration and active pursuit. Future studies will be necessary to validate the ability of inflammation to stimulate *de novo* lesion development, which will require use of novel mouse models and careful experimental design. However, analysis of existing patient records may yield indirect evidence of inflammation during disease progression. One such study tracked symptom onset in a small population of patients and found that over 60% of patients presented with symptoms during the fall and winter flu season, suggesting the possibility that sub-clinical viral infection could create the appropriate cellular context for disease progression (Flemming et al., 2015). Alternatively, the authors of this study suggested that the correlation could be caused by vitamin D deficiency, a potent antioxidant, during cloudy winter months, particularly intriguing given that vitamin D reduces lesion burden in mouse models of CCM (Gibson et al., 2015). Regardless of the cause, it is clear that there is still much to ascertain regarding the contribution of inflammation to CCM, with the hope that eventually the great catalog of anti-inflammatory drugs could be mined for new treatments for CCM.

## 6 Role of angiogenesis in CCM pathogenesis

Inflammatory stimulation, particularly chronic inflammation, is intimately linked with angiogenesis in the adult. Angiogenesis, the process of forming new vessels from pre-existing vessels, occurs in many tissues in response to hypoxia or tissue injury. Sprouting angiogenesis occurs in response to angiogenic growth factors (VEGF, bFGF, etc.), and requires the remodeling of the endothelial cytoskeleton, cell-matrix adhesion, and cell-cell contacts (for review, see (Kushner and Bautch, 2013)). Inflammatory cytokines can up regulate pro-angiogenic signaling pathways and stimulate endothelial proliferation. In addition, increased permeability is considered a precursor to angiogenesis and is activated by pro-angiogenic signals (Nagy et al., 2008). Disassembly of AJs, such as after endothelial exposure to activated neutrophils, is accompanied by the release of β−catenin from cell junctions (Potter et al., 2005, Tinsley et al., 2002) and is required for angiogenesis and vascular remodeling (Carmeliet et al., 1999, Wallez and Huber, 2008).

As regulators of endothelial AJ stability, CCM proteins are poised to be key negative regulators of angiogenesis, which has been closely examined in multiple studies. KRIT1, CCM2, and PDCD10 are required for vascular development, as homozygous deletion of each of these proteins causes embryonic lethality between E8.5 (KRIT1, CCM2) and E12.5 (PDCD10) (Chan et al., 2011, Whitehead et al., 2004). The severity of the defects in these animals makes it difficult to determine where and when in vascular development CCM proteins are critical. However in zebrafish, mutation or down-regulation of KRIT1 or CCM2 expression causes increased sprouting and failed lumenization of somatic vessels in addition to a dilated heart phenotype (Hogan et al., 2008, Liu et al., 2010, Whitehead et al., 2009). In contrast, loss of CCM3 causes malformation and dilation of newly formed cerebral vessels, but not heart dilation (Schleider et al., 2011). *In vitro*, studies using isolated endothelial cells have yielded somewhat contradictory findings. While some studies have shown that loss of KRIT1 or CCM2 inhibit the ability of human umbilical vein endothelial cells (HUVEC) to form tube-like structures (Liu et al., 2010, Whitehead et al., 2009, Zhu et al., 2010), others have shown that loss of KRIT1 causes increased endothelial sprouting, proliferation and migration (Wustehube et al., 2010). Knockdown of PDCD10 however, appears to consistently increase tube formation in both HUVEC and human brain microvascular endothelial cells (Schleider et al., 2011, Zhu et al., 2010). PDCD10 is also important for endothelial cell polarization and lumen formation through its interactions with members of the STRIPAK (striatin-interacting phosphatase and kinase) complex, including the GCKIII kinases (STK24, STK25, and Mst4) and PP2A phosphatase (Chan et al., 2011, Zheng et al., 2010), further linking PDCD10 with vascular development.

While these studies support the idea that CCM proteins are important regulators of angiogenesis, many of these studies failed to address the mechanism for CCM protein involvement in angiogenesis. Nonetheless, increased expression of several pro-angiogenic growth factors have been found in human CCM lesions, including VEGF, PDGF, and TGF-β (Abe et al., 2009, Jung et al., 2003, Maiuri et al., 2006, Yildirim et al., 2010), and increased VEGF expression has also been measured in KRIT1 deficient animals (Corr et al., 2012). DiStefano et al. showed that loss of KRIT1 increases VEGF expression by increasing nuclear β-catenin (DiStefano et al., 2014), where β-catenin stimulates transcription of VEGF (Glading and Ginsberg, 2010). The increase in VEGF expression leads to autocrine binding of VEGFR2 and subsequent activation of downstream angiogenic signaling pathways, suggesting that VEGF expression contributes to a feed-forward pathway promoting the CCM pathologic phenotype. Intriguingly, this study also pointed out that PDCD10, but not CCM2, also increases β-catenin transcriptional activity and VEGF expression (DiStefano et al., 2014). However, PDCD10 deficient cells reportedly exhibit a loss of VEGFR2 signaling and are unable to respond to VEGF stimulation (He et al., 2010). Alternatively, both KRIT1 and PDCD10 deficient animals exhibit activation of TGFβ/BMP signaling downstream of β-catenin, which promotes EndMT, altered endothelial polarity, and abnormal vessel formation (Guan and Couldwell, 2013, Lampugnani et al., 2010, Maddaluno et al., 2013). Blocking TGF-β signaling or β-catenin signaling can reduce CCM-like lesion formation in KRIT1 and PDCD10 deficient mouse models, respectively (Bravi et al., 2015, Guan and Couldwell, 2013, Lampugnani et al., 2010, Maddaluno et al., 2013). Wustehube et al. demonstrated that down-regulating KRIT1 expression suppressed Dll4 and the expression of Notch-dependent genes, increasing angiogenic sprouting, and that conversely over-expression of KRIT1 up-regulated Notch activity (Wustehube et al., 2010). Deficient Notch activity has also been seen in the absence of PDCD10 (You et al., 2013). However, no studies have examined Notch signaling in the absence of CCM2, thus it remains unclear whether all members of the CCM family ubiquitously regulate this pathway.

Taken together, this data strongly suggests that the CCM complex is a key regulator of angiogenesis as well as postulates a role for angiogenesis in the formation of CCM lesions. The formation of new lesions, may occur via a hijacking of the angiogenic process in which new vessel formation is subverted to form dilated cavernous vessels. The increase in expression of angiogenic growth factors in human CCM patients, and the correlation of VEGF expression with disease progression, certainly supports the idea that these mechanisms may be active during CCM formation. However, the work done to date has not defined exactly how this complex regulates the angiogenic process. More studies will be required to sort out the relative contributions of KRIT1, CCM2, and PDCD10 to the angiogenic process, and of neo-angiogenesis to the etiology of CCM.

## 7 Interplay between oxidative stress and inflammation in CCM disease: towards a comprehensive and integrated model of pathogenesis and therapeutic approaches

Because of the diversity of molecules and molecular mechanisms that can be modulated by CCM proteins, it is not a surprise that these proteins influence almost all aspects of endothelial biology, including vascular development, maintenance of endothelial cell homeostasis, and barrier function (Corr et al., 2012, Glading et al., 2007, Maddaluno et al., 2013, Whitehead et al., 2009, Zheng et al., 2010). On the other hand, the emerging pleiotropic functions of CCM proteins in the modulation of various redox-sensitive signaling pathways, and the accumulating evidence that oxidative stress, inflammation and angiogenesis may contribute significantly to the onset, progression and severity of CCM disease raise the question as to whether the underlying mechanisms are interconnected.

Consistent with this possibility, growing evidence demonstrates that indeed an intricate, reciprocal relationship exists between these processes, especially in human pathologies (Kim and Byzova, 2014, Kim et al., 2013b). Specifically, there is clear evidence that ROS and oxidative stress originating from various sources, including cell exposure to chemicals, drugs, or other agents that alter the cellular redox status, can lead to inflammation by activating a variety of redox-sensitive transcription factors, including NF-κB, AP-1, PPAR-γ, and HIF-1α, which drive the expression of pro-inflammatory genes, leading to induction of various cytokines and chemokines, such as Cell Adhesion Molecules, MCP-1, TNF-α, IL-1, and TGF-β (Kim et al., 2013b, Miller et al., 2010, Reuter et al., 2010). Moreover, ROS can also induce post-translational modification of proteins involved in important redox-sensitive signaling transduction pathways that modulate inflammatory and angiogenic responses, including Src, Ras, RhoA/RhoA kinase (ROCK), PI3 K, VEGF, and MAPK pathways (Ferro et al., 2012, Goitre et al., 2012, Ushio-Fukai and Nakamura, 2008). Conversely, production of ROS can result from activation of immune and endothelial cells by pro-inflammatory stimuli (Basuroy et al., 2009, Kim et al., 2013b). Indeed, although not viewed as typical immunogenic cells, endothelial cells are suggested to be sentinel cells that detect danger signals, initiate innate immune responses, produce pro-inflammatory cytokines and chemokines, and recruit immune cells. Such increase in the endothelial innate immunity has emerged as an important mechanism underlying the interplay among oxidative stress, inflammation and endothelial dysfunction (Chen et al., 2015). In addition, besides the well-established mechanistic connection between inflammation and angiogenesis, there is also clear evidence that ROS may promote angiogenesis, either directly or via the generation of active oxidation products, including peroxidized lipids (Kim and Byzova, 2014). Accordingly, ROS have been implicated both in physiological and pathological angiogenesis, and shown to modulate vascular remodeling and inflammatory responses (Chen et al., 2015, Fukai and Ushio-Fukai, 2011, Miller et al., 2010, Ushio-Fukai, 2009, Ushio-Fukai and Nakamura, 2008). Remarkably, experimental support that synergistic oxidative stress and inflammatory responses are indeed implicated in CCM disease pathogenesis was provided by the finding that the redox-sensitive up-regulation of c-Jun induced by KRIT1 loss-of-function was accompanied by the induction of COX-2, a major inflammatory mediator involved in vascular dysfunction (Goitre et al., 2014) (see Section 3). Consistently, whereas a close relationship between oxidative stress and local inflammation has been shown to underlie vascular disease of diverse etiology (Basuroy et al., 2009, Faraci, 2011, Kim et al., 2013b, Miller et al., 2010), accumulating evidence suggests that both oxidative stress (Gibson et al., 2015, Goitre et al., 2010, Goitre et al., 2014) and inflammation (Corr et al., 2012, Shenkar et al., 2007, Shi et al., 2009, Shi et al., 2016) play a critical role in CCM disease pathogenesis. On the other hand, it has been also suggested that neoangiogenic events may be necessary to cause CCM disease (Boulday et al., 2011). Taken together with the established molecular links and the emerging reciprocity between oxidative stress, inflammation and angiogenesis (Kim and Byzova, 2014, Kim et al., 2013b, West et al., 2010), these observations point to multifactorial and interconnected mechanisms whereby oxidative stress, inflammation and angiogenesis play key synergistic roles in CCM pathogenesis, with oxidative stress having potential primacy (Goitre et al., 2014). In accord with this latter possibility, recent evidence in animal models has suggested that oxidative stress may play an even more critical role in CCM disease than previously described due to systemic effects (Gibson et al., 2015). Finally, the recent findings that genetic variants of modifier genes related to oxidative stress and inflammatory responses are associated with phenotypic markers of CCM disease severity, suggest that inter-individual variability in susceptibility to either oxidative stress or inflammation may contribute to CCM disease pathogenesis (Choquet et al., 2014b, Choquet et al., 2016).

The potential existence of a unifying mechanism that connects both the pleiotropic functions and the dysfunctional effects of CCM proteins has clearly emerged from recent discoveries in cellular and animal models and surgical CCM samples. In particular, the discovery that defective autophagy is a common feature of loss-of-function of the three known CCM genes and may underlie the major phenotypic hallmarks of CCM disease suggests a potential unifying pathogenic mechanism that reconciles both the broad spectrum of signaling abnormalities and consequent pathologic effects linked to CCM protein dysfunctions, and the distinct therapeutic approaches proposed so far (Marchi et al., 2015, Marchi et al., 2016a, Marchi et al., 2016b). Indeed, most of the reported pleiotropic functions and dysfunctional effects of CCM proteins are directly or indirectly related to autophagy and its tight interconnection with redox homeostasis and signaling (Filomeni et al., 2015, Marchi et al., 2016b, Scherz-Shouval and Elazar, 2011), suggesting that the modulation of autophagy may represent an underlying and unifying mechanism for CCM protein physio-pathological functions. Consistently, autophagy is a converging point of multiple physiological and pathological pathways and may exert pleiotropic effects on several molecular and cellular processes (Choi et al., 2013). Specifically, autophagy plays a pivotal role in various signaling pathways linked to CCM proteins, including pathways involving Sirt1/FoxO1 (Liu et al., 2015, Zhao et al., 2010), JNK/c-Jun (Yogev and Shaulian, 2010), RhoA/ROCK (Mleczak et al., 2013), β-catenin (Lin et al., 2015), TGF-β (Marchi et al., 2015), MEKK3 (Linares et al., 2015), and KLF2/4 (Guixe-Muntet et al., 2016, Liao et al., 2015). Moreover, most phenotypic hallmarks of CCM disease can be linked to autophagic dysfunctions, including enhanced ROS production (Huang et al., 2011, Marchi et al., 2015) and EndMT (Marchi et al., 2015), as well as altered cell adhesion and angiogenic potential (Kim et al., 2013a, Kumar et al., 2013, Tuloup-Minguez et al., 2013), and enhanced endothelial cell sensitivity to oxidant-induced injury (Liu et al., 2015). Furthermore, recent insights point toward an important role of autophagy in the endothelial protective effects of several drugs known to confer vasoprotection, including simvastatin, vitamin D and other potential therapeutic compounds proposed so far for CCM disease prevention and treatment (Andres et al., 2014, Guixe-Muntet et al., 2016, Hoyer-Hansen et al., 2010, Lin et al., 2013, Marchi et al., 2016a, Marchi et al., 2016b). On the other hand, it is now becoming clear that autophagy and major antioxidant pathways act in concert to combat the deleterious effects of intracellular redox imbalance (Dodson et al., 2015). Indeed, the autophagy-lysosomal pathway is particularly important in protecting endothelial cells against redox stress-induced proteotoxicity, since it can degrade redox-damaged proteins without causing aberrant changes to the redox network needed for metabolism or signaling (Dodson et al., 2015). Moreover, accumulating evidence demonstrates that the autophagy pathway and proteins play a major role in a wide array of vascular processes, including angiogenesis and the paracrine regulation of vasoactive substances from the endothelium (Nussenzweig et al., 2015), as well as in balancing the beneficial and detrimental effects of immunity and inflammation (Kuballa et al., 2012, Levine et al., 2011), thereby contributing to protect against both abnormal angiogenic responses and infectious, autoimmune and inflammatory diseases. Thus, autophagy constitutes also a major candidate mechanism for mediating the crosstalk between oxidative stress, inflammation and angiogenesis, and integrating consequent cell responses in health and disease.

In this light, it is also possible to provide an alternative interpretation for recent findings suggesting that CCM arise from endothelial gain of MEKK3-KLF2/4 signaling (Cuttano et al., 2016, Zhou et al., 2016). Indeed, it is possible to envisage a hypothetical mechanistic model whereby the upregulation of the MEKK3-KLF2/4 signaling occurring upon loss-of-function of CCM proteins could be part of an abnormal adaptive response consequent to autophagic defects and increased ROS levels associated with this loss. Consistently, growing evidence suggests that MEKK3, KLF2 and KLF4 play a major role in endothelial redox homeostasis by acting both as upstream regulators and downstream targets of redox signaling (Chen et al., 2015, Hamik and Jain, 2012, Irani, 2011, Marin et al., 2013, Son et al., 2011) and autophagy (Guixe-Muntet et al., 2016, Guo et al., 2013, Liao et al., 2015, Linares et al., 2015). Specifically, whereas it has been recently reported that crosstalk between autophagy and KLF2 determines endothelial cell phenotype and microvascular function (Guixe-Muntet et al., 2016), there is evidence that KLF4 is a nodal transcriptional regulator of mitochondrial homeostasis and clearance via regulation of autophagy (Liao et al., 2015). On the other hand, it is known that the expression of these cytoprotective transcription factors is modulated by stimuli that induce oxidative stress and inflammatory responses in endothelial cells, such as disturbed flow and oxidized lipids, and is tightly regulated by redox-sensitive pathways acting at multiple levels, including transcriptional, posttranscriptional, and posttranslational. In turn, these KLFs regulate other redox-sensitive pathways to ultimately modulate the oxidative and inflammatory state of the endothelium, suggesting that they are involved in a complex and fine-tuned network of redox signaling that coordinates cellular responses to oxidative stress and inflammatory stimuli (Hamik and Jain, 2012, Irani, 2011, Marin et al., 2013). Thus, it is possibly that the recently reported causative relationship between the hyperexpression of two normally cytoprotective transcription factors, such as KLF2 and KLF4, and CCM pathogenesis (Cuttano et al., 2016, Zhou et al., 2016) could be connected to autophagic defects and oxidative stress events associated with loss-of-function of CCM proteins. Accordingly, growing evidence suggests that autophagic defects and elevation of intracellular ROS above a critical threshold may lead to the persistent hyperactivation of transcription factors known to play a crucial role in redox homeostasis maintenance and cell defense against oxidative stress, including NRF2 and a member of the KLF family, resulting in an abnormal cellular stress response that paradoxically reduces oxidative stress resistance leading to disease (Komatsu and Ichimura, 2010, Zucker et al., 2014). More in general, consistent with the dual physiological and pathological activities of redox-sensitive KLFs, it is now well established that in all biological systems, including vascular tissues, redox reactions bear the Janus faceted feature of promoting both physiological signaling responses and pathophysiological cues, as well as that endogenous antioxidant molecules and mechanisms participate in both scenarios (Dodson et al., 2015, Espinosa-Diez et al., 2015, Holmstrom and Finkel, 2014). Indeed, emerging evidence demonstrates that only intermediate levels of major regulators of antioxidant responses are beneficial, although both the low and high concentration thresholds for physiological vs. pathological effects may vary largely depending on genetic and environmental factors, and the cellular context (Dodson et al., 2015). Taken together with these considerations, the alternative interpretation provided above could also explain the apparent paradox that both loss (Fisher et al., 2015) and gain (Zhou et al., 2016) of MEKK3 function result in similar effects, including increased Rho/ROCK activity, impairment of neurovascular integrity, and brain blood vessels leakage, while only intermediate levels seem to be beneficial for the homeostasis of brain vasculature (Zhou et al., 2016).

Future studies are required to pave the way along the novel and intricate research avenues traced out by the potential interconnections among recent findings, including the emerging evidence that a fine-tuned crosstalk between redox signaling and autophagy plays a pivotal role in modulating both the pleiotropic functions of CCM proteins and the interplay between oxidative stress, inflammation and angiogenesis in CCM disease pathogenesis. Nonetheless, the novel therapeutic perspectives that recent scientific discoveries are opening up justify the commitment to address these difficult but exciting new research challenges.

## 8 Concluding remarks

The scientific advances and breakthroughs into the physiopathological functions of CCM proteins outlined in this review bring into view a new mechanistic landscape in which defective autophagy and altered redox signaling emerge as the major molecular mechanisms that underlie the pleiotropic effects of CCM protein dysfunctions, whereas oxidative stress and inflammation loom large as the key pathogenic determinants of CCM disease onset and progression. Consistently, emerging evidence suggests that autophagy, oxidative stress and inflammation are inextricably linked, and play major roles in the onset and progression of other vascular diseases (Choi et al., 2013, Nakahira et al., 2014, Ryter et al., 2010, Scherz-Shouval and Elazar, 2011, Vindis, 2015). Furthermore, it is clearly established that oxidative stress and inflammatory events have a focal nature and can indeed occur locally in the brain vasculature, triggering focal endothelial dysfunction in genetically predisposed blood vessels (Faraci, 2011). To this regard, it is noteworthy that recent population-based cohort studies have suggested that CCM disease pathogenesis involves an intricate interplay between multiple factors, including genetic and environmental risk factors associated with enhanced susceptibility to oxidative stress and inflammation (Choquet et al., 2014a, Choquet et al., 2014b, Choquet et al., 2016, Trapani and Retta, 2015). Indeed, besides providing further support to the important role of oxidative stress and inflammation in the onset and progression of CCM disease, this evidence suggests also a potential explanation for the heterogeneity described among affected individuals.

In view of this new mechanistic scenario, it is also possible to speculate that the loss-of-function of CCM genes may affect mainly low-blood-flow capillary beds due to their enhanced susceptibility to oxidative stress events. Consistently, it is now well understood that endothelial cells display remarkable heterogeneity in structure and function (Aird, 2012, Garlanda and Dejana, 1997), and respond differently to different profiles of hemodynamic forces, including induction of either antioxidant or pro-oxidant responses by high and low shear stress, respectively (Heo et al., 2011, White and Frangos, 2007). Indeed, the molecular basis of endothelial cell heterogeneity and the mechanotransduction mechanisms underlying endothelium's ability to discriminate between flow profiles are beginning to be elucidated (Heo et al., 2011, Li et al., 2005, Nigro et al., 2011, Nolan et al., 2013). On the other hand, it is becoming clear that there is a multiplicity of factors inherent to the physiopathology of NVUs and brain microvascular endothelial cells that increase opportunities for local oxidative stress events (Freeman and Keller, 2012). Together these mechanisms may plausibly explain both the focal nature of CCM lesions and the large inter-individual variability in CCM disease onset, progression, and severity. Indeed, the focal nature of CCM lesions has been a consistent observation in both naturally-occurring and experimental CCM, although the local determinants of focal lesion development have so far remained elusive or merely justified by the Knudson's genetic two-hit hypothesis. In particular, the possibility that local oxidative stress and inflammation events are indeed the key focal determinants of lesion development and may act in concert to drive CCM pathogenesis can explain the clear discrepancy between the pan-endothelial loss of CCM proteins and the focal nature of CCM lesions in neonatal mouse models (Boulday et al., 2011, Chan et al., 2011). Intriguingly, there is evidence that oxidative stress occurs after normal birth (Wilhelm et al., 2016), as well as that the immature brain is particularly susceptible to free radical injury because of its poorly developed scavenging systems and high availability of iron for the catalytic formation of free radicals (Blomgren and Hagberg, 2006). Besides providing further support to the role of oxidative stress in CCM pathogenesis, these observations could also explain why the formation of CCM-like lesions in neonatal mouse models of CCM disease occur only if the endothelial-specific deletion of CCM genes is induced immediately after birth (P1) but not in the adult phase (Boulday et al., 2011). Furthermore, there is evidence that cerebral endothelial cells contain a relatively high density of mitochondria, which may in part reflect the high metabolic demands of maintaining the BBB (Oldendorf et al., 1977), suggesting that mitochondrial dysfunction could have a particularly relevant impact on the brain vasculature. Taken together with the findings that mitochondrial dysfunction, increased ROS production and impairment of autophagy are major hallmarks of loss-of-function of CCM proteins (Goitre et al., 2010, Marchi et al., 2015), this evidence provides another plausible explanation to the preferential occurrence of CCM lesions in the brain.

Finally, the mechanistic landscape outlined in this review brings to the forefront distinct therapeutic perspectives, barely dreamt a decade ago, which now seem legitimately attainable. However, given that most of the emerging potential therapeutic compounds influence redox-sensitive mechanisms, both their low and high concentration thresholds for physiological vs. pathological effects should be carefully considered. Indeed, the existence of a physiological role of specific ROS concentrations can explain some of the negative results from clinical trials in which large doses of exogenously administered antioxidants or hyperactivation of antioxidant pathways with electrophilic therapeutics failed to improve outcomes of vascular diseases or resulted even deleterious (Dodson et al., 2015, Johansen et al., 2005).

In conclusion, this review highlighted current evidence for a major involvement of oxidative stress and inflammation in CCM disease, arguing that the interplay between these potential determinants should be considered and examined using an integrative systemic approach in order to better understand the complexity of the underlying molecular mechanisms. Such an approach may provide novel valuable insights into CCM pathogenesis, facilitating the development of novel targeted, safe and effective therapeutic strategies to counteract disease progression and clinical severity, including drug combination and personalized medicine strategies.

## Figures and Tables

**Fig. 2:**
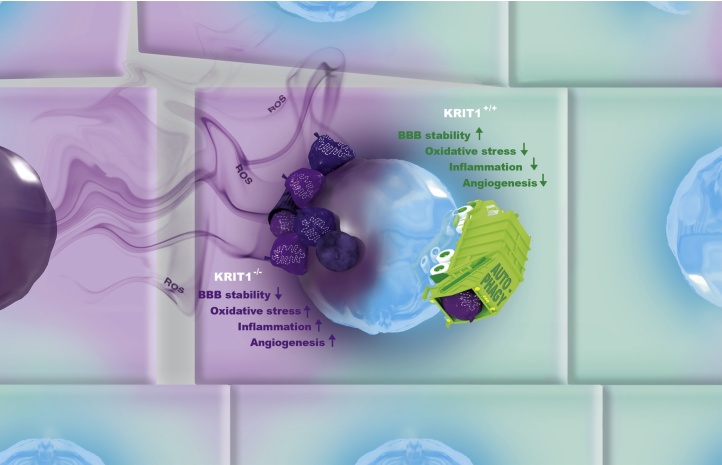
Schematic model of the relationship between loss-of function of a CCM protein and major phenotypes associated with CCM disease—Toward a unifying mechanistic scenario for CCM disease pathogenesis and treatment. CCM proteins play pleiotropic roles in distinct redox-sensitive pathways by modulating the fine-tuned crosstalk between redox signaling and autophagy. Effective autophagy removes ROS-generating cellular trash, including damaged mitochondria, to rejuvenate cell environment, thus serving a cytoprotective function for the maintenance of endothelial cell monolayer integrity and functionality and BBB stability even under adverse stress conditions. Loss-of-function of a CCM protein causes defective autophagy and altered redox signaling, affecting BBB stability and sensitizing endothelial cells to local oxidative stress and inflammatory events, which may act as key pathogenic determinants of focal formation and progression of CCM lesions. The common capacity to modulate the interplay between autophagy and redox signaling reconciles the distinct pharmacological approaches proposed so far for CCM disease prevention and treatment.

**Table 1:** Molecular pathways altered by loss-of-function of CCM proteins, and potential pharmacological approaches for the prevention or treatment of CCM disease proposed so far.

Molecular Pathway	KRIT1	CCM2	PDCD10	Pharmacological approaches	Experimental Articles
Adherens junctions	Yes	Yes	n.d.	Antioxidant compounds and autophagy inducers (Tempol, Vitamin D3); inhibitors of the TGF-β and β-catenin pathways (Sulindac sulfide and its analogs)	Glading et al., 2007, Glading and Ginsberg, 2010, Lampugnani et al., 2010, Corr et al., 2012, Gibson et al., 2015, Bravi et al., 2015, Bravi et al., 2016
Autophagy	Yes	Yes	Yes	Autophagy inducers (Rapamycin, Torin1)	Marchi et al., 2015
β-catenin*	Yes	No	Yes	Inhibitors of the β-catenin pathway (Sulindac sulfide and its analogs)	Glading and Ginsberg, 2010, Boulday et al., 2011, Bravi et al., 2015
β1 integrin adhesion	Yes	Yes	n.d.		Zhang et al., 2001, Zawistowski et al., 2002, Faurobert et al., 2013, Liu et al., 2013, Macek Jilkova et al., 2014, Renz et al., 2015
JNK/c-Jun	Yes	n.d.	n.d.	Antioxidant compounds (*N*-acetylcysteine)	Goitre et al., 2014
FoxO1	Yes	Yes	n.d.	Antioxidant compounds (*N*-acetylcysteine, Avenanthramide)	Goitre et al., 2010, Moglia et al., 2015, Gibson et al., 2015
Kruppel-like factors (KLF2/4)	Yes	n.d.	n.d.		Cuttano et al., 2016, Renz et al., 2015; Zhou et al., 2015*;*Zhou et al., 2016
MEKK3	Yes	Yes	Yes		Uhlik et al., 2003, Fisher et al., 2015; Zhou et al., 2015*;*Cullere et al., 2015, Zhou et al., 2016
Notch/ERK	Yes	n.d.	Yes	Multikinase inhibitors (Sorafenib)	Schulz et al., 2015, Wustehube et al., 2010, You et al., 2013
RhoA/ROCK	Yes	Yes	Yes	Inhibitors of Rho signaling and multi-target compounds (Statins, Fasudil, Tempol, vitamin D3)	Crose et al., 2009, Whitehead et al., 2009, Borikova et al., 2010, Stockton et al., 2010, Zheng et al., 2010, Chan et al., 2011, McDonald et al., 2012, Richardson et al., 2013, Gibson et al., 2015, Zhou et al., 2016
ROS**	Yes	Yes	Yes	Antioxidant compounds and autophagy inducers (*N*-acetylcysteine, Avenanthramide, Tempol, Vitamin D3, Torin 1, Pt NPs)	Goitre et al., 2010, Goitre et al., 2014, Fidalgo et al., 2012, Moglia et al., 2015, Gibson et al., 2015, Marchi et al., 2015, Moglianetti et al., 2016
STRIPAK***	No	No	Yes		Zheng et al., 2010, Chan et al., 2011, Fidalgo et al., 2012, Voss et al., 2007, Zhang et al., 2013b
TGFβ/BMP and EndMT	Yes	n.d.	Yes	Inhibitors of the TGF-β pathway (Sulindac sulfide and its analogs)	Maddaluno et al., 2013, Bravi et al., 2015, Bravi et al., 2016
VEGF	Yes	n.d.	Yes	VEGFR inhibitors (Semaxanib)	He et al., 2010, Zhu et al., 2010, DiStefano et al., 2014

n.d.—not determined.

* β-catenin transcriptional activity.

** ROS production and redox signaling.

*** STRIPAK (striatin-interacting phosphatase and kinase) complex-related pathways.
